# Collateral Tissue Damage by Several Types of Coagulation (Monopolar, Bipolar, Cold Plasma and Ultrasonic) in a Minimally Invasive, Perfused Liver Model

**DOI:** 10.5402/2011/518924

**Published:** 2011-07-18

**Authors:** Thomas Carus, Klaas Rackebrandt

**Affiliations:** ^1^Department of General, Visceral and Vascular Surgery, Center of Minimally Invasive Surgery, Hospital Cuxhaven—University of Hanover Teaching Hospital, 27474 Cuxhaven, Germany; ^2^Medical Engineering, Bremerhaven University of Applied Sciences, 27568 Bremerhaven, Germany

## Abstract

Hemostasis in minimally invasive surgery causes tissue damage. Regardless of the method of production of thermal energy, a quick and safe coagulation is essential for its clinical use. In this study we examined the tissue damage in the isolated perfused pig liver using monopolar, bipolar, cold plasma, and ultrasonic coagulation. In a minimally invasive in vitro setup, a 2-3 cm slice of the edge of the perfused pig liver was resected. After hemostasis was achieved, liver tissue of the coagulated area was given to histopathological examination. The depth of tissue necrosis, the height of tissue loss, and the time until sufficient hemostasis was reached were analyzed. The lowest risk for extensive tissue damage could be shown for the bipolar technique, combined with the highest efficiency in hemostasis. Using cold plasma, coagulation time was longer with a deeper tissue damage. Monopolar technique showed the worst results with the highest tissue damage and a long coagulation time. Ultrasonic coagulation was not useful for coagulation of large bleeding areas. In summary, bipolar technique led to less tissue damage and best coagulation results in our minimally invasive model. These results could be important to recommend bipolar coagulation for clinical use in minimally invasive surgery.

## 1. Introduction

A safe and efficient hemostasis is one of the most important factors in minimally invasive surgery. Hemostasis can be achieved by sutures, clips, application of hemostyptica or thermic coagulation [1]. 

For thermic coagulation, high-frequency (HF) coagulation in monopolar or bipolar mode, cold plasma, and ultrasonic coagulation are used [2–4]. In addition to hemostasis all techniques cause tissue damage [5]. 

The different techniques have not yet been compared in terms of tissue damage. Klingele compared the ultrasonic technique with the bipolar technique in dissected vessels and examined the tissue damage [6].

Campagnacci et al. and Underwood et al. measured the differences in operative time, blood loss and complications when using bipolar or ultrasonic technique in colectomies and fundoplications [7, 8]. The tissue damage was not described. Sutton et al. examined the lateral thermal spread using monopolar and bipolar diathermy, the Harmonic scalpel and LigaSure by measuring the maximum temperature [9].

In our present study, four different types of coagulation technique were examined. The tests were performed on perfused, isolated pig liver in a minimally invasive model with a Pulsatile Organ Perfusion (P.O.P.) trainer. For analysis more than 1,500 histopathological data were collected.

## 2. Methods

### 2.1. Experimental Setup

**Figure 1:**
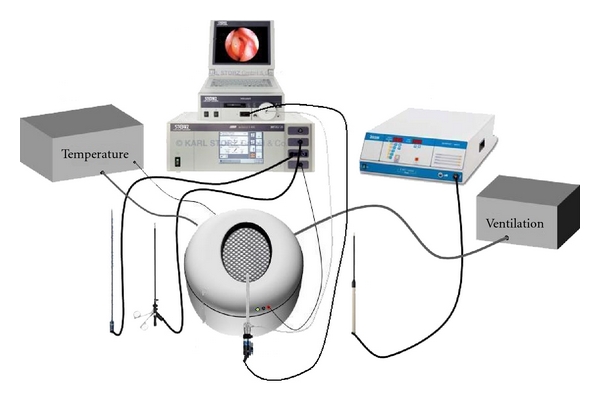
Laparoscopic equipment and P.O.P. trainer.

A complete laparoscopic equipment setup for minimally invasive surgery was used together with a P.O.P. trainer (Figure 1). 

The experimental procedure began with heating the isolated, frozen liver in a water-cooled vacuum in order to warm it up to about 30°C–35°C before the actual start of the experiment. This reduced the time for the subsequent warming to body temperature (37°C). The P.O.P. trainer was filled with colored saline solution and heated to 37°C.

**Figure 2:**
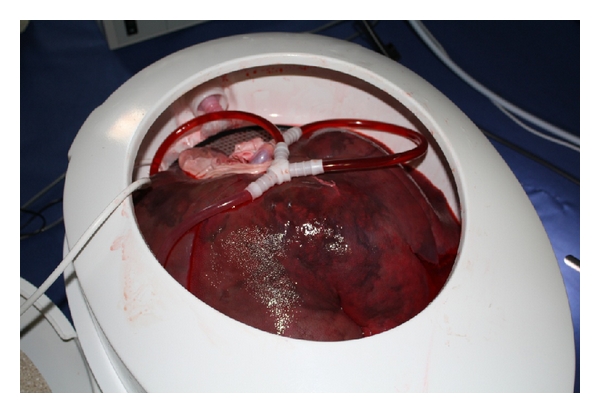
Isolated, pulsatile perfused pig liver.

Hepatic artery and portal vein were connected with the corresponding adapters using surgical sutures. The adapters were connected by hoses with the P.O.P. trainer. The pump of the trainer was activated to check the correct function (Figure 2). Small open vessels on the liver surface were closed with sutures. After a final check of the correct connection of blood vessels, the unit was closed with a cover. After 30 minutes, the tissue temperature was detected by the laboratory thermometer at 5-minute intervals until it reached 37°C. 

**Figure 3:**
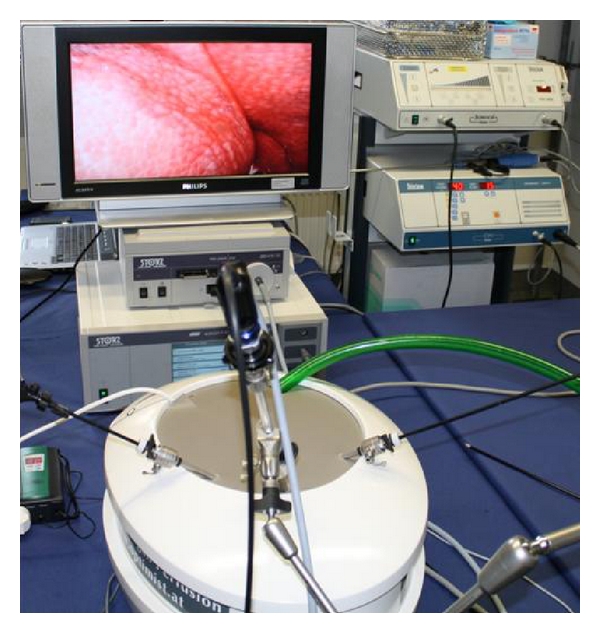
Laparoscopic setup with liver bleeding before coagulation.

To start the experiment, the pump was stopped and the cover of the P.O.P. trainer was removed. With a scalpel, a 2-3 cm thick slice of the liver was resected to create a deep tissue bleeding. After closure of the cover, the pump was activated again; the bleeding area was photodocumented (Figure 3). 

**Figure 4:**
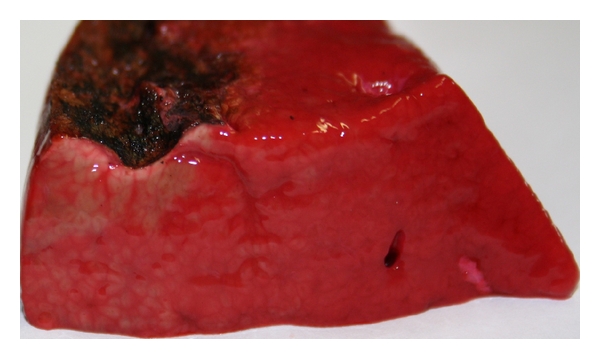
Liver tissue after coagulation of the upper half.

The cut surface was divided horizontally in two halves. Only the upper half was coagulated to see the difference to untreated tissue and calculate the tissue loss (Figure 4). The coagulation was started using one of the four techniques until the bleeding could be stopped. 

Using ultrasonic coagulation, it was not possible to create hemostasis in preliminary experiments for *hemostasis of large bleeding areas* in perfused pig liver.

**Figure 5:**
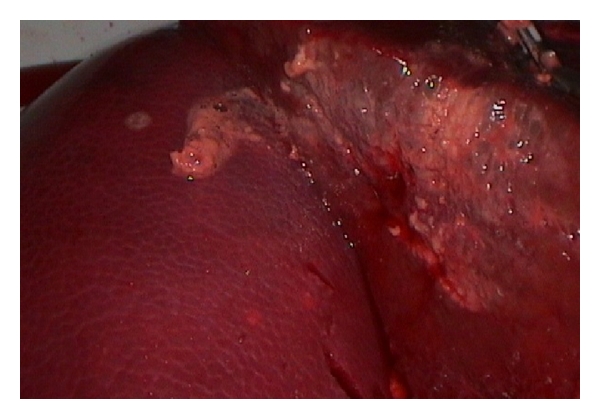
Liver tissue after 5-min ultrasonic coagulation.

**Figure 6:**
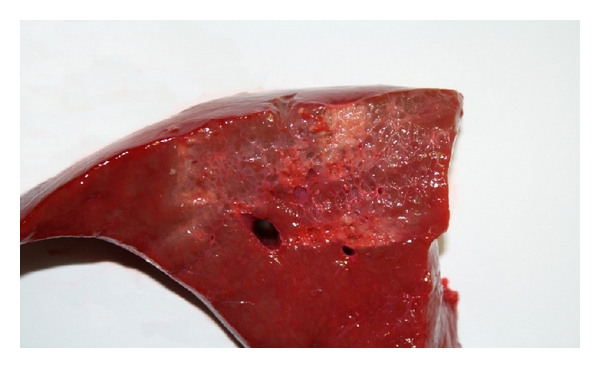
Liver tissue after 15-min ultrasonic coagulation.

After 5 minutes, the cell walls were highlighted and a first approach of coagulated tissue became visible. The bleeding did not stop; the extraction of tissue by the mechanical vibrations of the ultrasonic applicator could be observed. After 10 minutes, there was an increase of foam; after 15 minutes, we saw extracted tissue without sufficient hemostasis (Figures 5 and 6). 

Based on these preliminary tests, the ultrasonic technique was not considered for further experiments and pathological examination. 

Using the other devices, after stopping the pump and opening the cover, the coagulated tissue was resected with a margin of 2 cm. The tissue was fixed on a cork plate and placed in formalin solution after photodocumentation. 

### 2.2. Histopathological Examination (Pathologic Institute Bremerhaven, Germany (Head Professor Dr. M. Heine))

**Figure 7:**
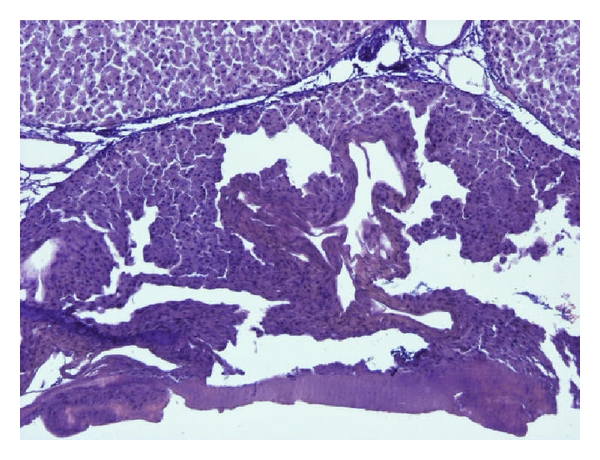
Tissue necrosis after bipolar coagulation.

From the resected tissue, 10 cuts in 5–10 mm distance were made, each cut was examined at 10 points microscopically (Figure 7). 100 values could be determined per day and method.

For analysis, the mean tissue injury was the key parameter. Furthermore, the maximum tissue damage was determined to compare the tissue loss caused by the different techniques.

### 2.3. Settings of the Coagulation Devices

The high-frequency coagulation devices were used with the settings shown in Table 1. These settings are similar to settings in minimally invasive, clinical use.

The gas flow had little effect (cooling) on the coagulation performance. With 1.5 L/min gas flow, the power output was comparable with the other techniques. For the bipolar coagulation “Bipolar Soft Coag” was used. In monopolar mode “Forced Coag” was chosen because the required output voltage of the monopolar technique for a similar coagulation is significantly higher than in bipolar technology. The mode used provided a comparable performance in accordance with a higher voltage.

### 2.4. Evaluation

**Figure 8:**
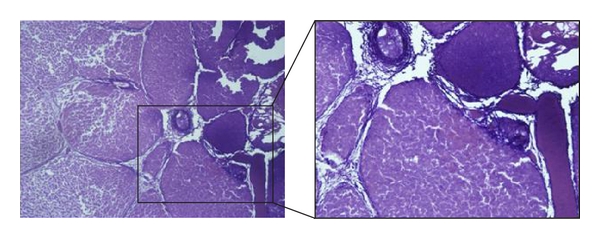
Microscopic margin between normal and necrotic cells.

Figure 8 illustrates the transition of the nonaffected tissue to necrotic tissue. The tissue structure changes on the necrotic side the color is darker in the thermally damaged area. 

Microscopically the necrotic cells are not clearly defined and shrank because of the evaporated water. In addition, the color is very dark compared with the nondamaged tissue. 

The tissue damage can be measured reproducibly with an accuracy of 0.05 mm. The 10 measuring points are lined up linearly, so that the measurements cover a distance of 1 mm of the tissue sample. For the task a one-dimensional analysis of the tissue damage is sufficient.

The tissue loss is measured in relation to the lower half of the untreated sample at the point of greatest loss. The coagulated tissue shows a darker color and shrinkage. The measurement can be easily carried out macroscopically (Figure 4). 

### 2.5. Statistical Analysis

For statistical evaluation of tissue damage and tissue loss, the SPSS software was used.

## 3. Results

### 3.1. Coagulation Time

We measured the coagulation time of 3 different techniques (monopolar, bipolar, and cold plasma) on 4 experimental days. Ultrasonic coagulation was excluded, because hemostasis of large bleeding areas could not be reached. 

**Figure 9:**
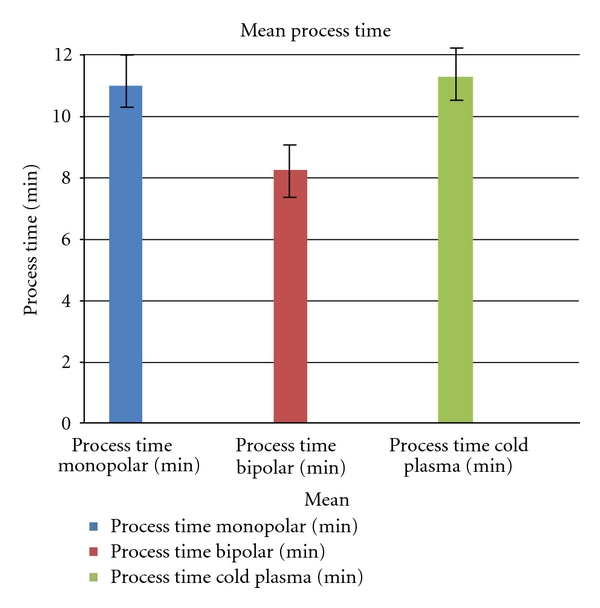
Coagulation time for hemostasis.

As shown in Figure 9, the shortest time was needed for bipolar coagulation (mean 8.25 min). There was almost no difference between monopolar (11 min) and cold plasma (11.25 min).

### 3.2. Tissue Loss

**Figure 10:**
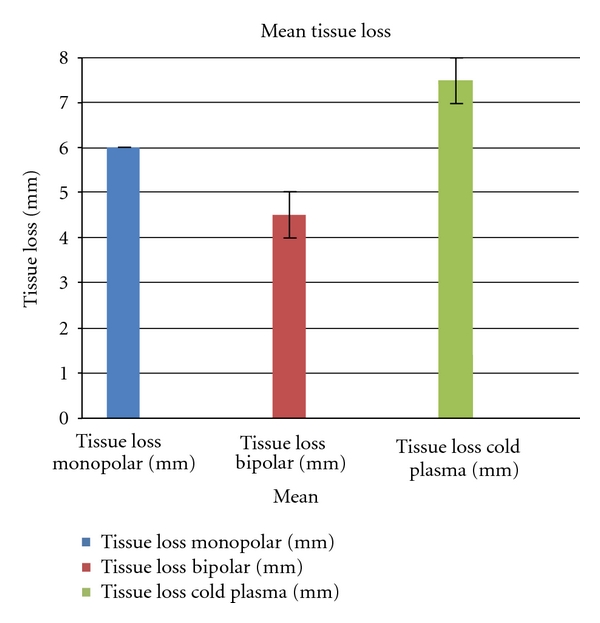
Mean tissue loss.

To determine the tissue loss, the height difference between the coagulated area and the untreated area was macroscopically analyzed and measured in mm (Figure 10).

The minimal tissue loss was 4 mm (bipolar coagulation); the maximal tissue loss of 8 mm occurred using cold plasma coagulation. On average, the tissue loss was the least in bipolar technique (4.5 mm), more in monopolar technique (6.0 mm), and most in cold plasma coagulation (7.5 mm).

### 3.3. Tissue Damage

**Figure 11:**
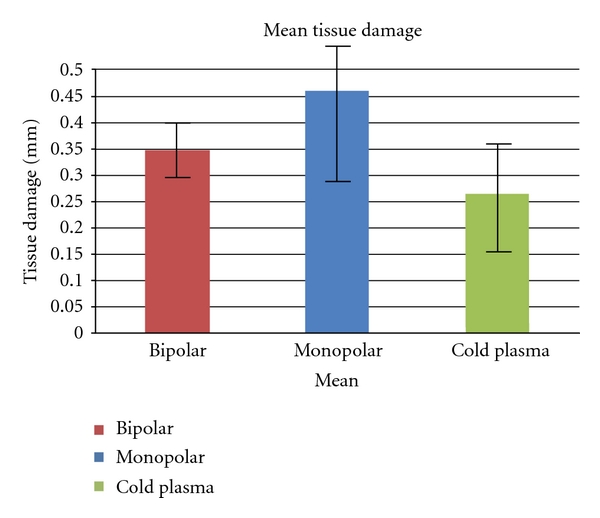
Mean tissue damage.

The depth of tissue necrosis was analyzed microscopically and measured in mm (Figure 11).

The lowest value was found in cold plasma coagulation (0.157 mm), the highest in monopolar coagulation (0.598 mm). The slightest average damage to the tissue was observed in cold plasma coagulation with a mean of 0.264 mm. Bipolar coagulation led to an average depth of 0.346 mm, monopolar coagulation of 0.459 mm.

### 3.4. Evaluation of the Results

Bipolar coagulation of large bleeding areas of the perfused pig liver led to a rapid hemostasis causing moderate tissue damage and depth loss. The worst results occurred with monopolar coagulation compared to bipolar technique (coagulation time + 33.3%, tissue loss + 33.3%, tissue damage + 32.7%). Ultrasonic coagulation was excluded because large bleeding areas could not be treated sufficiently and hemostasis was not reached.

## 4. Discussion

The aim of this study was to examine whether there are differences between the common coagulation techniques in a minimally invasive model concerning the efficiency of hemostasis and local tissue damage [1, 3]. The experiments were done with isolated, perfused pig livers.

Monopolar coagulation, bipolar coagulation and cold plasma coagulation, were used. The use of ultrasonic coagulation did not lead to a complete hemostasis even after 15-minute coagulation time. The termination resulted from the application form of energy causing foam formation and extraction of tissue components. Therefore, this technique was excluded from our study.

The benefits of ultrasonic technology in minimally invasive surgery are clear in the preparation and dissection of tissue, the use close to other organs, and the closure of isolated vessels of up to 5–7 mm. In our model, it was not useful for coagulation of large bleeding areas.

The first parameter in this study was the coagulation time until a sufficient hemostasis was reached. A good instrument should be safe and quick to reduce damage to lateral tissue and to minimize the energy flow to the patient. This as well reduces the operation time and the costs.

Bipolar coagulation was the fastest technique and needed 33% less time than monopolar coagulation. This correlated with the second parameter—the tissue loss in the coagulated area. We know from clinical use that with longer monopolar coagulation time more and more necrotic tissue sticks to the instrument and is pulled out. This leads to new bleeding points and extends the coagulation time. 

The average tissue loss was 4.5 mm in bipolar technique and 6.0 mm in monopolar technique (cold plasma coagulation 7.5 mm).

**Figure 12:**
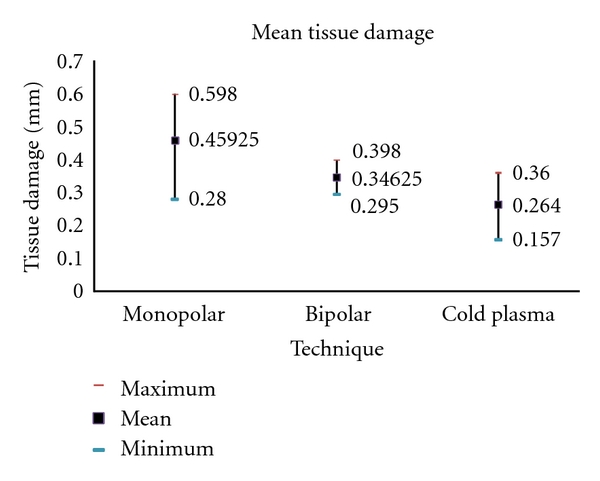
Statistical analysis of the tissue damage.

The depth of tissue damage by coagulation was the third parameter in this study. Because of the high efficiency of bipolar coagulation, less energy was needed to stop the bleeding. This resulted in an average depth of tissue necrosis of 0.346 mm in bipolar coagulation and 0.459 mm in monopolar coagulation. The difference was statistically significant with *P* < 0.05 (Figure 12).

**Figure 13:**
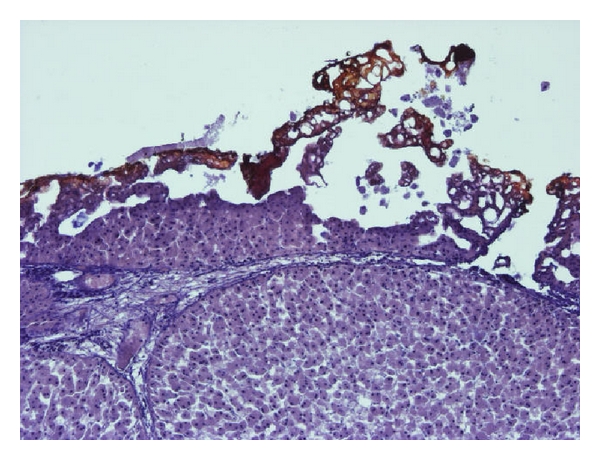
Microscopic analysis after cold plasma coagulation.

The cold plasma coagulation as the only contactless technique has a special position. It showed the longest coagulation time and the highest tissue loss, while the depth of tissue damage was the lowest in this study. One possible explanation could be the gas, which is pressed into the tissue during the contactless coagulation. It leads to a deep dissection of necrotic tissue and a rapid shrinkage of the particles (Figure 13).

**Figure 14:**
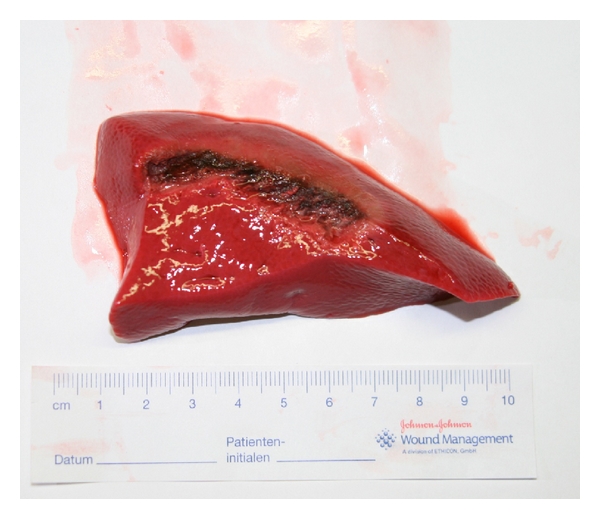
Liver tissue after bipolar coagulation.

Macroscopic examination of the coagulated tissue reveals differences between the techniques. After bipolar coagulation the tissue has a red to brownish color. The supplied energy led to a protein denaturation without carbonization. This indicates a low degree of necrosis and corresponds with the previously mentioned results (Figure 14).

**Figure 15:**
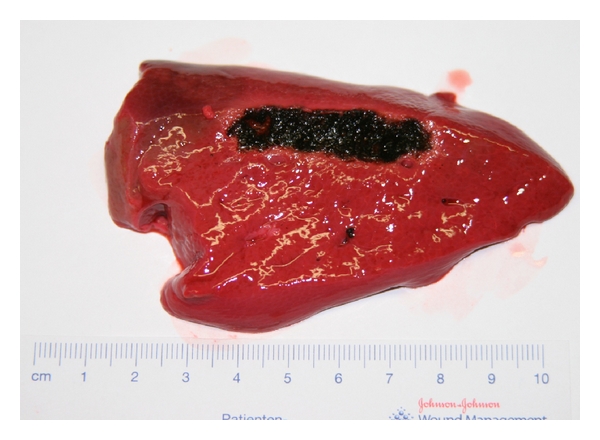
Liver tissue after monopolar coagulation.

**Figure 16:**
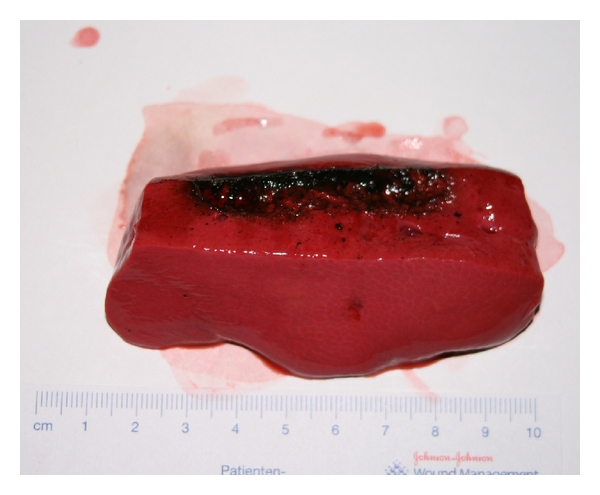
Liver tissue after cold plasma coagulation.

After monopolar coagulation, we regularly see black carbonized tissue (Figure 15), after cold plasma coagulation grey-brown to black tissue (Figure 16).

In summary, the collateral tissue damage consisted of the tissue loss and the tissue necrosis in the depth. When both factors are added, the total damage can be calculated (Table 2).

Bipolar coagulation technique shows essential advantages compared with bipolar and cold plasma coagulation.

The results of this work came from experiments on isolated pig livers in an in vitro test environment and cannot be easily transferred to human application. Perfusion of the isolated liver was achieved with colored saline solution and not with blood, what restricts hemostasis and prolongs the coagulation time. This is one reason for the relatively long coagulation time that we measured in the perfused liver. Further studies have to show the presumed superiority of bipolar technique.

In our surgical department, we do not use monopolar coagulation in the abdominal cavity. Ultrasonic technique is used for preparation and dissection, bipolar technique for coagulation [1].

## 5. Conclusions

The experimental study showed differences between several coagulation techniques in a minimally invasive model for hemostasis in deep liver bleeding. Hemostasis could be reached by monopolar, bipolar, and cold plasma coagulation. Ultrasonic coagulation, which is very useful in dissection of tissue and coagulation of isolated vessels, did not lead to a sufficient hemostasis in large bleeding areas.

The bipolar technique was the form of coagulation with the highest efficiency and the least tissue damage. These results could be important for clinical use too.

##  Disclosure

The authors confirm that no financial relationships exist in relation to this work.

## Figures and Tables

**Table 1:** Settings of the high-frequency coagulation devices.

Technique	Power
Monopolar	40 watt level 3
Bipolar	40 watt level 3
Cold plasma	40 watt 1.5 L/min
Ultrasonic	High level

**Table 2:** Calculation of the total damage.

	Tissue damage (mm)	Tissue loss (mm)	Total damage (mm)
Monopolar	0.43	6.0	6.43
Bipolar	0.35	4.5*	4.85*
Cold plasma	0.16	7.5	7.66

*P < 0.05.
